# PD1/PD-L1 targeting in advanced soft-tissue sarcomas: a pooled analysis of phase II trials

**DOI:** 10.1186/s13045-020-00891-5

**Published:** 2020-05-19

**Authors:** Antoine Italiano, Carine Bellera, Sandra D’Angelo

**Affiliations:** 1 Early Phase Trials and Sarcoma Units, Institut Bergonié, 229 Cours de l’Argonne, Bordeaux, France; 2 University of Bordeaux, Bordeaux, France; 3 University of Bordeaux, Inserm, Bordeaux Population Health Research Center, Epicene team, UMR 1219, F-33000 Bordeaux, France; 4 Inserm CIC1401, Clinical and Epidemiological Research Unit, Institut Bergonié, Comprehensive Cancer Center, F-33000 Bordeaux, France; 5 Memorial Sloan Kettering Cancer Center, New York, NY USA; 6 Weill Cornell Medical College, New York, NY USA

**Keywords:** Sarcoma, Immunotherapy, Pooled analysis

## Abstract

Immune checkpoint inhibitors, especially the programmed cell death receptor-1/ligand 1 (PD-1/L1) inhibitors, displayed promising efficacy in several solid tumor types and hematological malignancies. Data related to their activity in soft-tissue sarcomas (STS) are scarce.

We performed a pooled analysis of clinical trials investigating a PD1 or PD-L1 antagonist in patients with advanced STS. Three hundred eighty-four patients were included in the pooled analysis; of those, 153 (39.8%) received a PD1/PD-L1 antagonist as a single agent. In patients treated with anti-PD1/PDL1 as a single agent, the overall response rate (ORR) and non-progression rate (NPR) were 15.1% and 58.5% respectively. In patients treated with a combination regimen, the ORR and NPR were 13.4% and 55.8% respectively. Analysis by histological subtype revealed that patients with alveolar soft part sarcoma and undifferentiated pleomorphic sarcoma exhibited the highest response rates and leiomyosarcoma the lowest. PD-L1 expression rate was low and inconsistently associated with objective response.

PD-1/PD-L1 antagonists have limited activity in unselected STS. Future studies should implement histology and immune-based stratification of STS in their design as well as sequential blood and tissue sampling to better understand the mechanisms of resistance and response given sarcomas inherent heterogeneity.

To the editor,

Several phase II studies investigating the activity of PD1/PD-L1 targeting in soft-tissue sarcoma (STS) patients have been recently reported. All of them were characterized by a limited sample size and high heterogeneity in terms of histological subtypes. Here, we report a pooled analysis of these studies in order to get insight on the global activity of PD1/PD-L1 in STS and according to histological subtype.

Trials investigating a PD1 or PD-L1 antagonist in patients with advanced STS were eligible for this pooled analysis if they were registered in a public trials’ registry. The efficacy results had to be published in English in a peer-reviewed journal or presented in Proffered paper sessions of an annual meeting of the American Association of Cancer Research, American Society of Clinical Oncology, Connective Tissue of Oncology Society, or European Society of Medical Oncology up to June 30, 2019.

Objective response (OR), stable disease (SD), progressive disease (PD), and non-evaluable (NE) patients were defined as per RECIST v1.1. Investigational treatment was described according to 2 (monotherapy vs. combination therapy) or 3 classes (monotherapy, combination of PD1/PD-L1 antagonist with another immunotherapy, or combination of PD1 or PD-L1 antagonist with another type of treatment). Main histological subtypes included undifferentiated pleomorphic sarcoma (UPS), liposarcoma (LPS), leiomyosarcoma (LMS), alveolar soft part sarcoma (ASPS), and other STS. Additional details are available in Supplementary Methods.

Nine multicenter clinical trials met eligibility criteria for this pooled analysis (supplementary Table 1). Overall, 384 patients were included; of those, 153 (39.8%) received a PD1 or PD-L1 antagonist as a single agent.

Overall, objective response rate (ORR) and non-progression rate (NPR) were 15.1% (95% CI [8.6%; 25.1%]) and 58.5% (95% CI [44.4%; 71.3%]) respectively (Table 1). In patients treated with anti-PD1 or anti-PDL1 as a single agent, the ORR and NPR were 18.7% (95% CI [2.1%; 71.6%]) and 63.6% (95% CI [25.3%; 90.0%]) respectively. In patients treated with a combination regimen, the ORR and NPR were 13.4% (95% CI [6.0%; 27.1%]) and 55.8% (95% CI [35.0%; 74.8%]) respectively. These results do not suggest an association between the ORR (or NPR) and the investigational strategy (anti-PD1 or anti-PDL1 used as a single agent or in combination with another immunomodulatory drug or another class of anti-cancer agent).

**Table 1:** Pooled analysis of efficacy of PD1/PD-L1 antagonist in soft-tissue sarcomas

All population (*n* = 384)					
		ORR		NPR	
		15.1		58.5	
		95% CI 8.6% ; 25.1%		95% CI 44.4% ; 71.3%	
Efficacy by histological subtype					
Histological subtype	*N* patients	ORR		NPR	
		%	95% CI	%	95% CI
UPS	103	15.7	7.5-30.0	50.5	39.0-61.9
LMS	82	6.9	2.0-21.3	54.1	29.3-77.0
DDLPS	61	7.3	1.2-33.7	54.5	24.5-81.6
ASPS	41	48.8	26.0-72.0	80.5	54.1-93.5
Others	97	10.3	5.0-20.2	52.1	35.5-68.3
Efficacy by the therapeutic strategy					
PD1/PD-L1 single agent	153	18.7	2.1-71.6	63.6	25.3-90.0
Combination with other immunotherapy	114	11.4	3.5-31.4	57.9	18.2-89.4
Combination with non-immunological agent	117	14.0	0.5-84.2	53.8	7.9-94.0

*ASPS* alveolar soft part sarcoma, *DDLPS* liposarcoma, *LMS* leiomyosarcoma, *ORR* objective response rate*, NPR* non-progression rate, *UPS* undifferentiated pleomorphic sarcoma

ORR and NPR were 15.7% (95% CI [7.5%; 30.0%]) and 50.5% (95% CI [39.0%; 61.9%]) for UPS, 7.3% (95% CI [1.2%; 33.7%]) and 54.5% (95% CI [24.5%; 81.6%]) for LPS, 6.9% (95% CI [2.0%; 21.3%]) and 54.1% (95% CI [29.3%; 77.0%]) for LMS, 48.8% (95% CI [26.0%; 72.0%]) and 80.5% (95% CI [54.1%; 93.5%]) for ASPS, and 10.3% (95% CI [5.0%; 20.2%]) and 52.1% (95% CI [35.5%; 68.3%]) for other sarcomas (supplementary table 2).

Three clinical trials reported data related to PD-L1 expression status. Overall PD-L1 expression (≥ 1%) in tumor cells was observed in 21 (13.6%) out of 154 patients with available data. Twenty of them were evaluable for response: 6 had an objective response for an ORR of 30% in PDL1-positive tumors. Among the 133 patients with PD-L1-negative status, nine had an objective response.

The low level of PD-L1 expression we have observed here is in agreement with previously reported retrospective studies using validated anti-PD-L1 immunohistochemical assays [1]. Although the proportion of objective responses is higher in patients with PD-L1-positive tumors, responses were also observed in PD-L1 negative cases. This highlights the limitation of PD-L1 expression as a predictive biomarker.

Data related to the genetic and immunologic landscape of STS are scarce. Pollack et al. reported a study investigating the immune phenotype of the most common individual STS subtypes in a series of 87 cases [2]. The authors found that UPS had the highest levels of PD-L1 and of PD-1 expression as well as the highest level of T cell infiltration in comparison with other histological subtypes. These results suggested that UPS were more likely to respond to immune checkpoint inhibitors and our pooled analysis confirmed this assumption.

Previous studies have already shown that LMS are poorly infiltrated by T cells [1]. Pre-existing T cell antitumour immunity has been hypothesized as a prerequisite to the anti-PD-1/PD-L1 response. Altogether, these results may explain the extremely low response rate to PD1/PDL1 inhibition observed in LMS and the need to investigate innovative strategies to modify the microenvironment of these tumors which are characterized by a strong infiltration by M2 macrophages [3].

We noted an ORR about 8% in DDLPS suggesting the need for alternative strategies to galvanize an immune response. CKD4 inhibitors have demonstrated some efficacy in DDLPS which are characterized by a strong CDK4 amplification [4, 5]. Combination of PD1/PD-L1 antagonists with such agents, which have been shown to enhance immunogenicity of tumor cells, can represent a potential promising approach [6].

Our pooled analysis suggests also that PD1/PD-L1 targeting may have also a role in translocation-associated sarcomas. Indeed, the highest objective response rate has been observed in ASPS with nearly 50% of patients having an objective response. However, the mechanisms which are driving the immunogenicity of this ultra-rare sarcoma remain to be elucidated.

Overall, this pooled analysis underscores the need for future studies implementing a histology and immune-based stratification of STS patients in their design. Petitprez et al. have reported an immune-based classification of complex genomics STS; transcriptomic profile of a cohort of 608 STS was performed utilizing the microenvironment cell populations-counter (MCP-counter) method [7]. Tumors were assigned to one of five sarcoma immune classes (SICs), labeled A, B, C, D, and E, with SIC A, “immune desert,” being characterized by the lowest expression of gene signatures related to immune cells and SIC E being characterized by the highest expression of genes specific of immune populations such as T cells, CD8+ T cells, NK cells, and cytotoxic lymphocytes. Interestingly, intra-tumoral tertiary lymphoid structures (TLS) were identified as a hallmark of the inflamed SIC E group [7]. There are accumulating evidence indicating that TLS play a crucial role in antitumor immune responses by favoring presentation of tumor antigens by dendritic cells and education of subsequent T and B cell responses, resulting in the production of T effector memory cells, memory B cells, and antibodies [8]. Their presence can be easily assessed by immunohistochemistry and has been associated with improved outcome in several tumor types [8]. Utilizing tumor specimens from SARC028, Petitprez et al. demonstrated that, when treated with pembrolizumab, patients with SIC E tumors, which are characterized by the presence of TLS, exhibit significantly higher ORR and progression-free survival than others [7]. Indeed, the objective response rate was 50% in SIC E patients versus 25% in SIC D and 22% in SIC, *p* = 0.026. In addition, CR were only found in SIC E and no responder was found in SIC A and B [7]. These data suggest that using TLS as a biomarker may represent a promising approach to tailor immunotherapy in sarcoma patients.

## Supplementary information

**Additional file 1.** Supplementary methods and results

## Data Availability

Not applicable.

## Abbreviations

NE: Non-evaluable
OR: Objective response
ORR: Overall response rate
PD: Progressive disease
PD1: Programmed cell death 1
PD-L1: Programmed death-ligand 1
SD: Stable disease
STS: Soft-tissue sarcoma
