# Supplementary material for: PD1/PD-L1 targeting in advanced soft-tissue sarcomas: a pooled analysis of phase II trials
Source: J Hematol Oncol. 2020 May 19;13:55. doi: 10.1186/s13045-020-00891-5 (PMC7236113; doi:10.1186/s13045-020-00891-5)
Supplement: Supplementary file 1 — Additional file 1. Supplementary methods and results [file 13045_2020_891_MOESM1_ESM.docx]

**SUPPLEMENTARY METHODS**

***Endpoints and Statistical Analysis***

For each trial, objective response rate (ORR) was defined as the number of responses (complete [CR] and partial [PR]) divided by the total number of patients (CR + PR + SD + PD + NE). Non-progression rate (NPR) was defined as the number of CR, PR and SD divided by the number of reported patients.

To account for varying sample sizes across trials, we relied on generalized linear models (binomial link function) to provide unbiased estimates of the trial-level ORR and NPR using a the trial as a random effect. We reported the estimated mean trial-level ORR and NPR, together with their respective 95% confidence intervals (95%CI). Statistical significance was set at 5%. Statistical analyses were performed using SAS version 9.4 software (SAS Institute, North Carolina, USA).

**SUPPLEMENTARY RESULTS**

| **Supplementary Table 1. Characteristics of the studies included in the pooled analysis (n=384)** | | | | | | | | | | | | | |
| --- | --- | --- | --- | --- | --- | --- | --- | --- | --- | --- | --- | --- | --- |
| **Therapeutic strategy** | **Investigational drug(s)** | **Reference** | **N patients** | **UPS** | | **LPS** | | **LMS** | | **ASPS** | | **OTHER** | |
|  |  |  |  | **N** | **%** | **N** | **%** | **N** | **%** | **N** | **%** | **N** | **%** |
| Monotherapy | Pembrolizumab | 1,2 | 98 | 40 | 40.8% | 34 | 34.7% | 10 | 10.2% | 0 | 0.0% | 14 | 14.3% |
| Monotherapy | Atezolizumab | 6 | 22 | 0 | 0.0% | 0 | 0.0% | 0 | 0.0% | 22 | 100% | 0 | 0% |
| Monotherapy | Nivolumab | 4 | 33 | 12 | 36.4% | 3 | 9.1% | 13 | 39.4% | 1 | 3.0% | 4 | 12.1% |
| Combination :  Immunotherapy + other | Pembrolizumab  plus doxorubicin | 8 | 36 | 3 | 8.3% | 4 | 11.1% | 10 | 27.8% | 0 | 0% | 19 | 52.8% |
| Combination :  Immunotherapy + other | Pembrolizumab +  cyclophosphamide | 5 | 47 | 16 | 34.0% | 2 | 4.3% | 15 | 31.9% | 0 | 0.0% | 14 | 29.8% |
| Combination :  Immunotherapy + other | Pembrolizumab +  axitinib | 7 | 34 | 5 | 14.7% | 2 | 5.9% | 6 | 17.7% | 12 | 35.3% | 9 | 26.5% |
| Combination :  Double immunotherapy | Pembrolizumab +  NKTR 214 | 9 | 40 | 10 | 25.0% | 10 | 25.0% | 10 | 25.0% | 0 | 0.0% | 10 | 25.0% |
| Combination :  Double immunotherapy | Durvalumab +  tremelimumab | 3 | 43 | 5 | 11.6% | 6 | 14.0% | 5 | 11.6% | 6 | 14.0% | 21 | 48.8% |
| Combination :  Double immunotherapy | Nivolumab +  Ipilimumab | 4 | 31 | 12 | 38.7% | 0 | 0.0% | 13 | 41.9% | 0 | 0% | 6 | 19.4% |

***Footnotes****: ASPS= alveolar soft-part sarcoma, LMS= Leiomyosarcoma, LPS= Liposarcoma, UPS= undifferentiated pleomorphic sarcoma*

| **Supplementary Table 2. Objective response rates according to histological subtypes and therapeutic strategy (n=384)** | | | | | | | | | | | |
| --- | --- | --- | --- | --- | --- | --- | --- | --- | --- | --- | --- |
| **Therapeutic strategy** | **N patients** | **UPS** | | **LPS** | | **LMS** | | **ASPS** | | **OTHER** | |
|  |  | **N** | **%** | **N** | **%** | **N** | **%** | **N** | **%** | **N** | **%** |
| Monotherapy | 153 | 52 | 19.2% | 37 | 8.1% | 23 | 4.3% | 23 | 43.5% | 18 | 11.1% |
| Combination :  Immunotherapy + other | 117 | 24 | 8.3% | 8 | 25% | 31 | 12.9% | 12 | 58.3% | 42 | 9.5% |
| Combination :  Double immunotherapy | 113 | 27 | 18.5% | 16 | 0% | 27 | 3.7% | 6 | 50% | 37 | 10.8% |

**REFERENCES**

1. Tawbi HA, Burgess M, Bolejack V, Van Tine BA, Schuetze SM, Hu J, D'Angelo S, Attia S, Riedel RF, Priebat DA, Movva S, Davis LE, Okuno SH, Reed DR, Crowley J, Butterfield LH, Salazar R, Rodriguez-Canales J, Lazar AJ, Wistuba II, Baker LH, Maki RG, Reinke D, Patel S. [Pembrolizumab in advanced soft-tissue sarcoma and bone sarcoma (SARC028): a multicentre, two-cohort, single-arm, open-label, phase 2 trial.](https://www.ncbi.nlm.nih.gov/pubmed/28988646) Lancet Oncol. 2017 Nov;18(11):1493-1501.

2. Burgess M, Bolejack V, Schuetze S, Van Tine B, Attia S, Riedel R, Hu J, Davis L, Okuno S, Priebat D, Movva S, Reed D, D'Angelo S, Lazar A, Keung E, Reinke D, Baker L, Maki R, Patel S, Tawbi H. [Clinical activity of pembrolizumab (P) in undifferentiated pleomorphic sarcoma (UPS) and dedifferentiated/pleomorphic liposarcoma (LPS): Final results of SARC028 expansion cohorts.](https://ascopubs.org/doi/abs/10.1200/JCO.2019.37.15_suppl.11015) J Clin Oncol 2019 37:15_suppl, 11015-11015

3 Somaiah N, Conley A, Lin H, Sanchez-Espiridion B, Amini B, Ravi V, Araujo D, Patel S, Benjamin R, Zarzour M, Sabir S, Wang WL, Lazar A, Wistuba I, Hwu P. A phase II multi-arm study to test the efficacy of durvalumab and tremelimumab in multiple sarcoma subtypes

4 D'Angelo SP, Mahoney MR, Van Tine BA, Atkins J, Milhem MM, Jahagirdar BN, Antonescu CR, Horvath E, Tap WD, Schwartz GK, Streicher H. [Nivolumab with or without ipilimumab treatment for metastatic sarcoma (Alliance A091401): two open-label, non-comparative, randomised, phase 2 trials.](https://www.ncbi.nlm.nih.gov/pubmed/29370992) Lancet Oncol. 2018;19:416-426.

5 Toulmonde M, Penel N, Adam J, Chevreau C, Blay JY, Le Cesne A, Bompas E, Piperno-Neumann S, Cousin S, Grellety T, Ryckewaert T, Bessede A, Ghiringhelli F, Pulido M, Italiano A. [Use of PD-1 Targeting, Macrophage Infiltration, and IDO Pathway Activation in Sarcomas: A Phase 2 Clinical Trial.](https://www.ncbi.nlm.nih.gov/pubmed/28662235) JAMA Oncol. 2018;4:93-97

6 Coyne G, Sharon E, Moore N, Meehan R, Takebe N, Juwara L, Rubinstein L, Read W, Riedel R, Merriam P, Hu J, Burgess M, Doroshow J, Chen A. Phase II study of atezolizumab in patients with alveolar soft part sarcoma. Connective Tissue Oncology Society 2018 Annual Meeting

7 Wilky BA, Trucco MM, Subhawong TK, Florou V, Park W, Kwon D, Wieder ED, Kolonias D, Rosenberg AE, Kerr DA, Sfakianaki E, Foley M, Merchan JR, Komanduri KV, Trent JC. [Axitinib plus pembrolizumab in patients with advanced sarcomas including alveolar soft-part sarcoma: a single-centre, single-arm, phase 2 trial.](https://www.ncbi.nlm.nih.gov/pubmed/31078463) Lancet Oncol. 2019 Jun;20(6):837-848.

8 Pollack S, Redman MW, Wagner M, Loggers E, Baker K, McDonnell S, Gregory J, Copeland V, Hammer K, Johnson R, Moore R, Shahnazari M, Townson S, Lewis Jones R, Cranmer L. [A phase I/II study of pembrolizumab (Pem) and doxorubicin (Dox) in treating patients with metastatic/unresectable sarcoma.](https://ascopubs.org/doi/abs/10.1200/JCO.2019.37.15_suppl.11009) J Clin Oncol 2019 37:15_suppl, 11009-11009

9 D'Angelo S, Conley A, Kelly C, Dickson M, Gounder M, Chi P, Keohan M, Livingston J, Patel S, Adamson T, Kiesler H, Biniakewitz M, Phelan H, Condy M, Agaram N, Qin LX, Erinjeri J, Hwang S, Tap W. [Pilot study of NKTR214 and nivolumab in patients with sarcomas.](https://ascopubs.org/doi/abs/10.1200/JCO.2019.37.15_suppl.11010) J Clin Oncol 2019 37:15_suppl, 11010-11010
